# A Computational Strategy to Select Optimized Protein Targets for Drug Development toward the Control of Cancer Diseases

**DOI:** 10.1371/journal.pone.0115054

**Published:** 2015-01-27

**Authors:** Nicolas Carels, Tatiana Tilli, Jack A. Tuszynski

**Affiliations:** 1 Laboratório de Modelagem de Sistemas Biológicos, National Institute of Science and Technology for Innovation in Neglected Diseases (INCT/IDN, CNPq), Centro de Desenvolvimento Tecnológico em Saúde, Fundação Oswaldo Cruz, Rio de Janeiro, Brazil; 2 Department of Oncology, Faculty of Medicine & Dentistry, University of Alberta, Edmonton, Alberta, Canada T6G 1Z2; 3 Department of Physics, University of Alberta, Edmonton, Alberta, Canada T6G 2E1; Rutgers—New Jersey Medical School, UNITED STATES

## Abstract

In this report, we describe a strategy for the optimized selection of protein targets suitable for drug development against neoplastic diseases taking the particular case of breast cancer as an example. We combined human interactome and transcriptome data from malignant and control cell lines because highly connected proteins that are up-regulated in malignant cell lines are expected to be suitable protein targets for chemotherapy with a lower rate of undesirable side effects. We normalized transcriptome data and applied a statistic treatment to objectively extract the sub-networks of down- and up-regulated genes whose proteins effectively interact. We chose the most connected ones that act as protein hubs, most being in the signaling network. We show that the protein targets effectively identified by the combination of protein connectivity and differential expression are known as suitable targets for the successful chemotherapy of breast cancer. Interestingly, we found additional proteins, not generally targeted by drug treatments, which might justify the extension of existing formulation by addition of inhibitors designed against these proteins with the consequence of improving therapeutic outcomes. The molecular alterations observed in breast cancer cell lines represent either driver events and/or driver pathways that are necessary for breast cancer development or progression. However, it is clear that signaling mechanisms of the luminal A, B and triple negative subtypes are different. Furthermore, the up- and down-regulated networks predicted subtype-specific drug targets and possible compensation circuits between up- and down-regulated genes. We believe these results may have significant clinical implications in the personalized treatment of cancer patients allowing an objective approach to the recycling of the arsenal of available drugs to the specific case of each breast cancer given their distinct qualitative and quantitative molecular traits.

## Introduction

Cancer is one of the most challenging and complex diseases worldwide. Although a significant improvement in diagnosis and treatment occurred in the past few years, cancer remains the leading cause of death worldwide, which is forecast to reach a staggering 13.2 million deaths by 2030 [1]. These numbers can only get worse as a result of the general trends of population aging and population growth [2].

Body cells may become cancerous as a result of genetic and epigenetic reprogramming processes involving complex regulatory circuits leading to their immortality and uncontrolled division [3]. The process of uncontrollable cellular division goes in parallel with an increase of tumor mass resulting in local physiological disturbances leading to metastases and ultimately the death of the whole organism often due to cachexia or organ failures. Metastases present the biggest challenge to medical management of cancer, being the main cause of death of cancer patients [4].

The identification of molecular mechanisms that drive tumorigenesis and cancer progression represents a critical step in providing more efficacious therapeutics, improved diagnostics, and in correlating clinical behavior with disease etiology. In the last three decades, hundreds of potential cancer-related molecular targets (oncotargets) have been identified and therapeutics developed aiming at these targets. Many of the currently available drugs designed for specific cancers are very expensive, provide modest improvements in overall survival, and have significant negative side-effects. The most critical question to address is the nature of the molecular targets that must be understood in order to control cancer cell proliferation with as minimal as possible side effects in order to maintain reasonable quality of life of the patient. Being a genetic dysregulation disease with wide-spread physiological consequences, cancer intrinsically requires administration of complex multi-drug therapeutics. The identification of suitable therapeutic targets for treatment with drug cocktails is not simple given that cancerous cells do not have obvious molecular structure differences when compared with normal cells. Actually, the differences between normal and malignant cells rather lie in their regulation [5], which can be evaluated through transcriptome data. The recent progress in *in silico* data mining and high throughput data generation relative to gene, protein and metabolic networks [6,7] offers a new very promising opportunity to identify those proteins that would be of marginal implications in normal cells, but would become signaling hubs in cancer cells because of their natural high rate of connectivity with other proteins and a significant modification of expression rates.

Complex networks are ubiquitous in physics, biology and social sciences. Mathematically, a network may be described by a directed or undirected graph G = (V, E) with vertex and edge sets V and E, respectively. An edge appears in the graph if there is a known interaction of the two partners, either by direct binding or by enzymatic catalysis. An automorphism is a permutation of the set V that preserves the adjacency relation and, if present, the orientation of arrows between vertices. With the operation of composition, the automorphisms form a group Aut(G). Recent work by MacArthur et al. [8] lists 20 examples of real world networks and their rich symmetry groups. Real networks display a modular structure, with vertices organized in communities tightly connected internally and loosely connected to each other [9]. This results in the presence of symmetric subgraphs such as trees and complete cliques, which help to classify the nodes of a network into a “backbone” (those that remain fixed under the automorphisms) and “appendages” (those that get mapped to other vertices).

Such a strategy has been investigated recently in several papers [10–12]. These authors showed that the probability of 5-year patient survival (data from the SEER database, http://seer.cancer.gov/) is inversely proportional to the complexity of the signaling network (taken from the Kyoto Encyclopedia of Genes and Genomes – KEGG, http://www.genome.jp/kegg/) for the types of cancer considered. The network’s complexity was described through the use of Shannon entropy by quantifying the distribution of connections in protein interactomes represented as oriented graphs. Thus, the complexity of a graph can be formulated in terms of network entropy H(G) by summing on nodes and considering d(v) as the degree of edges v and defining H(G) = − Σ d(v) log d(v). Signaling pathways and networks drive both normal physiological and pathological processes in cells. A method to identify and delineate these signaling pathways would be useful to design new drugs for future cancer therapy development. To obtain further insights into the underlying mechanisms of breast cancer signaling pathways, we evaluated gene expression patterns for a number of breast cell lines in order to generate cell-line-specific network. Here, we search for protein targets with significant number of connections that are differentially expressed in several malignant cell lines of breast in relation to a non-tumoral cell line. We found that several putative proteins with large *connectivity* are not differently expressed when considering malignant and normal cell lines and, thus, cannot be considered for drug development without significant deleterious collateral effects for the patient’s health. However, another set of proteins with large *connectivity* are, indeed, down- or up-regulated in malignant cell lines and the up-regulated ones are potential targets for drug development. By analyzing the literature, we could verify that all of the putative protein targets obtained through our analysis are well known, and some, have already been used as agents for cancer control through drug therapy. However, other potential targets emerged from this study that are not used as drug targets yet, which raises the hypothesis that these putative targets might motivate specific drug development to increase the efficiency of existing drug cocktails. We found both similarities and significant differences when comparing the lists of top-ranked targets for different breast cancer cell lines. As a consequence, a specific cocktail might be considered in the context of *personalized medicine* according to the particular set of putative targets identified in the biopsy of a given patient. We propose that such personal assistance is expected to significantly increase the efficacy of cancer treatment.

## Results

**Figure 1:**
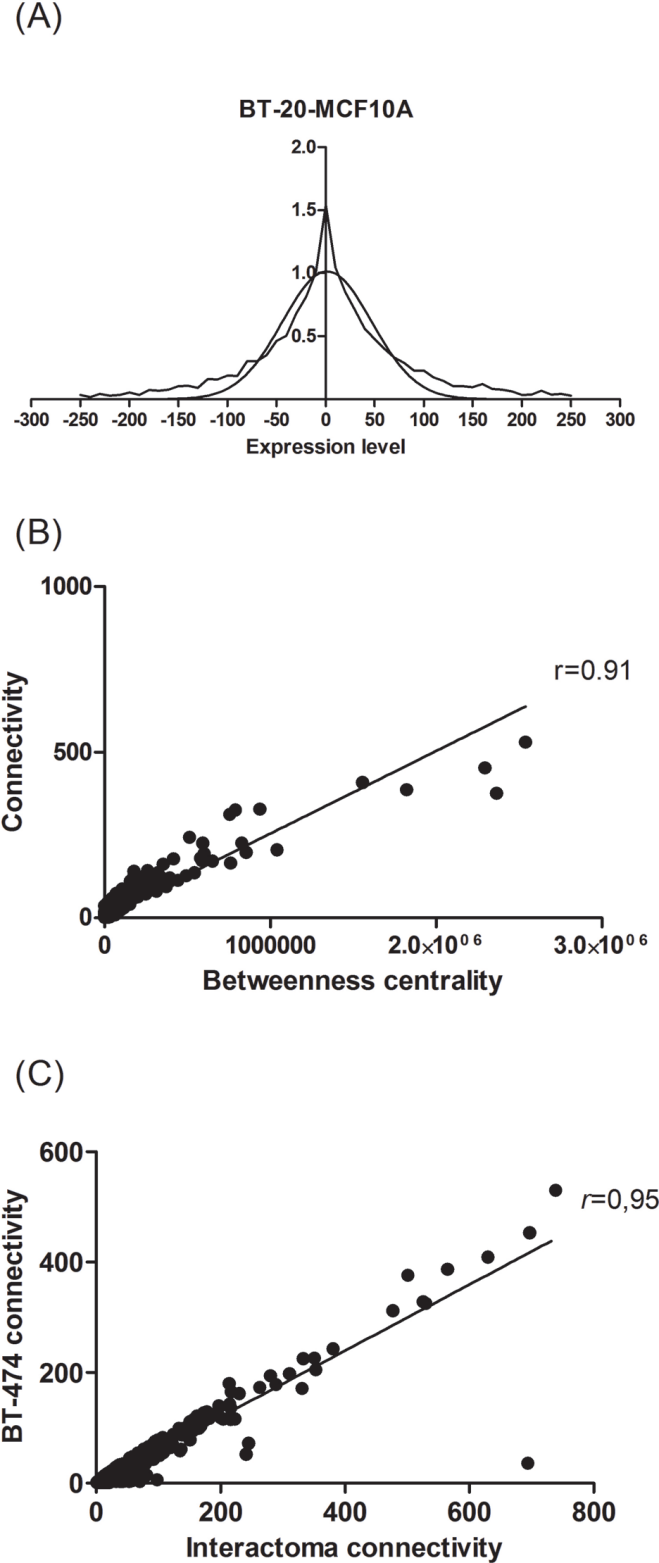
(A) Gaussian distribution of differential expressed genes between BT-20 and MCF10A. (B) Correlation between *connectivity* and *betweenness centrality*. (C) Correlation between the connectivity of proteins considering the full network sample available (∼10,000) and sub-networks of ∼600 proteins in BT-474 breast cancer cell lines.

The normalization of tag samples according to CDS size and tag number resulted in values of gene expression that may differ from one sample to the other just because of sample size. Such a trivial bias was successfully eliminated by using *Q*-norm over all samples analyzed in this study. The distribution of tag counts from transcriptome data is typically a decreasing curve where the lowest expressed genes are the most frequent ones. The subtraction of normalized tag counts of each malignant cell line from the normal cell line MCF10A gave frequency distributions whose shape was very narrow, but symmetric and centered on zero. The log_10_(*x*_i_+1) transformation together with the *Q*- norm resulted in symmetrical distributions (observed) very close to a Gaussian distribution (theoretical) (see Fig. 1A). With the theoretical distribution in hand, we calculated the classification thresholds of −90 and +90 tags on the observed distribution corresponding to a *p*-value α = 5% on the theoretical distribution. The classification thresholds corresponding to a *p*-value α = 1‰ were −150 and +150, respectively. Thus, the genes of malignant cell were classified as down-regulated when their tag count was lower than −90 or −150 or as up-regulated when their tag count was larger than +90 or +150 according to α = 5% and α = 1‰, respectively, by comparison to MCF10A.

Protein hubs are defined as proteins (nodes) with a much larger connection number (edges) than the average values in a protein (or gene) network; they act as global signal integrators or global regulators for multiple signaling pathways. When considering betweenness centrality and protein connectivity, we found a large positive correlation (*r* = 0.91) on a sample of ∼10,000 interacting proteins. Because connectivity is an objective measure that is simpler to calculate than betweenness centrality, we will only consider protein connectivity below (see Fig. 1B). We also found a positive correlation (*r* = 0.95) between the connectivity of proteins considering the full network sample available (∼10,000) and sub-networks of ∼600 proteins (see Fig. 1C). Thus, we considered the protein connectivity at a level of the sub-network as representative of the full network, which allowed the expression of protein connectivity as a relative value, which is rather robust to sample size variations.

**Figure 2:**
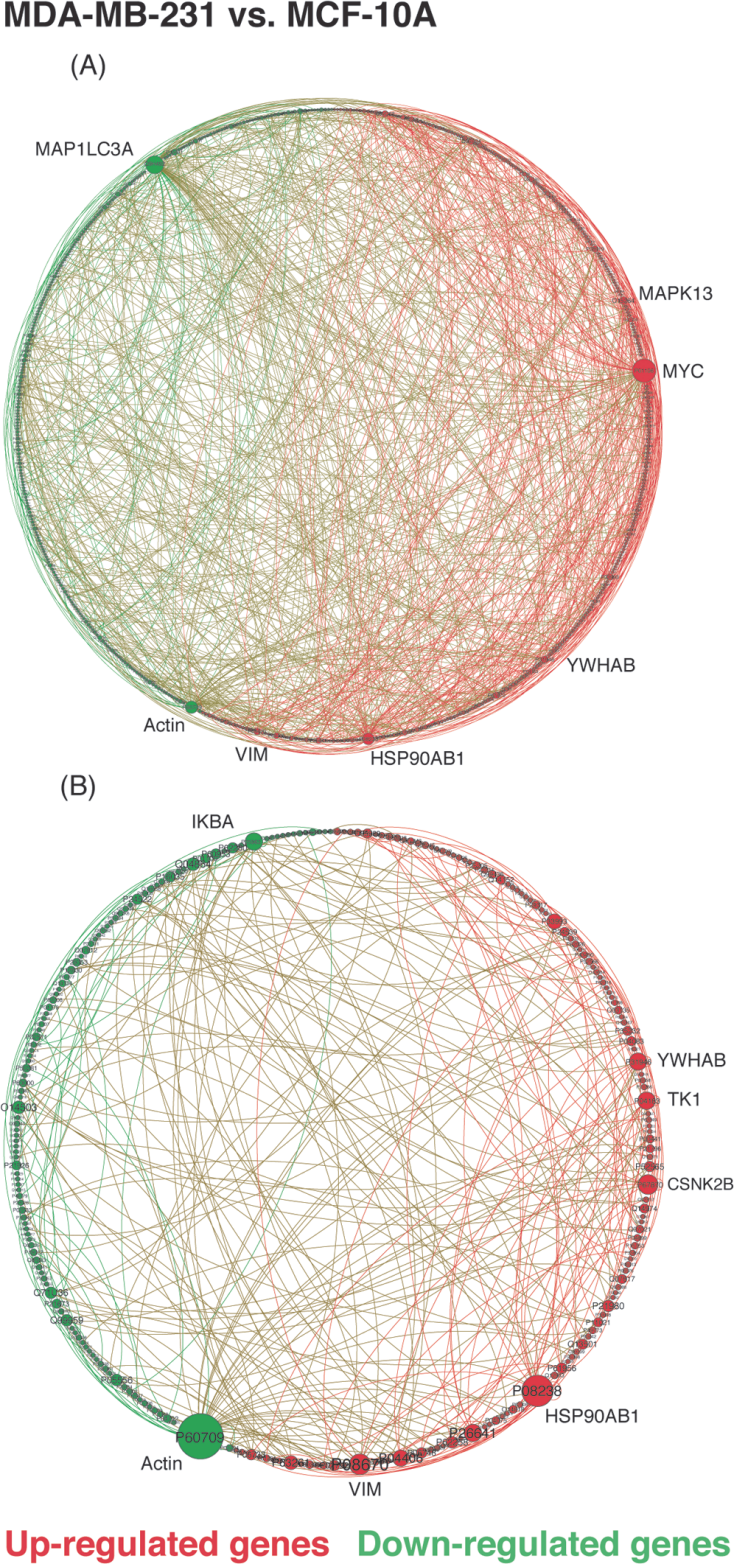
Sub-network of differentially expressed genes between MDA-MB-231 (Triple-Negative) and MCF10A, represented in a circular layout. Nodes represent genes while links represent interaction between genes. Size nodes indicate connectivity and color represents an expression pattern between tumoral versus non-tumoral breast cell line. (A) p<0.05. (B) p<0.01. Gephi was used to present and visualize the networks.

A map of network interactions between down- and up-regulated genes in breast malignant cell lines is given in Fig. 2. The top 5 most connected genes in sub-networks of up- and down-regulated genes are suitable candidates as protein targets for drug development (see S1 and S2 Tables).

**Figure 3:**
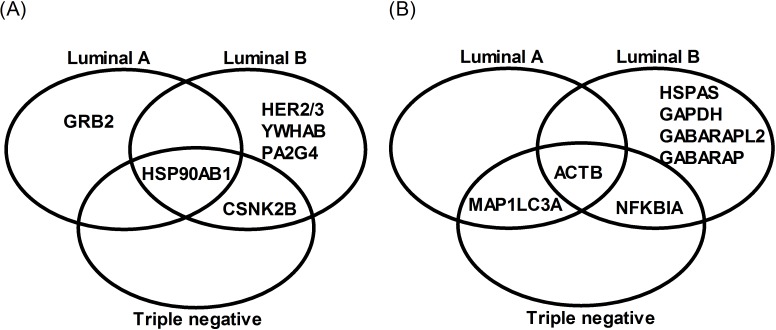
Venn diagram including comparative analysis of the subtype-specific networks to predict subtype-specific therapy. Up-regulated genes (A) and down- regulated genes (B). Luminal A classification includes MCF-7, T47D e ZR751 cell lines; Luminal B, includes BT-474; and triple negative, BT-20, MDA-MB-231 and MDA-MB-468.

The Venn diagram in Fig. 3A and B shows a subset of genes that were differentially expressed in each histological subtype in relation to control cells. Among up-regulated genes, we identified HSP90AB1 as a protein hub that is up-regulated in all malignant cells and is reported to induce angiogenesis. Interestingly, we found CSNK2B as a protein hub that is up-regulated in luminal B and triple negative malignant cell lines, but usually not in luminal A cells. GRB2 and HER2/3, YWHAB, PA2G4 are specifically up-regulated in luminal A and B, respectively. Actin is a protein hub that is down-regulated in all histological subtypes. We identified down-regulation of VIM in luminal A and B; NFKBIA between triple negative and luminal B; and MAP1LC3A in luminal A and triple negative. HSPAS, GAPDH, GABARAPL2 and GABARAP are down-regulated in luminal B.

To search for putative druggable oncoproteins in breast cancer, we focused here, on up-regulated genes. Our investigation revealed oncotargets (top 5-genes) related to cell cycle control, resisting cell death, inducing angiogenesis, invasion and metastasis, deregulating cellular energetics, genome instability and mutation, and tumor-promoting inflammation, which are hallmarks of cancer [3]. In triple negative subtype and luminal A, a higher percentage of up-regulated genes are related to sustaining proliferative signaling, resisting cell death, and activating invasion and metastasis. By contrast, in luminal B, up-regulated genes are preferentially associated to angiogenesis induction, cell death resistance, and invasion and metastasis activation (see S3 Table) [3,13–41].

In triple negative tumors that generally present poor prognosis, we observed the up-regulation of genes involved in (i) cell cycle control, which include EGFR, MAPK13, YWHAB, MAGOH, EEF1G, CSNK2B, MYC, SRPK1, TK1, GABARAPL1, and CHD3; (ii) anti-apoptotic factors such as YWHAB, MYC, GABARAPL1, and HDGF; and (iii) activation of invasion and metastasis such as YWHAB, SRPK1, CSNK2B, GABARAPL1, CHD3, and HDGF. The most significant up-regulated transcripts were those related to the deregulation of cellular energetics (GAPDH); this emerging hallmark was only observed in the triple negative subtype. This hallmark is also related to MYC and GABARAPL1. Only 1 transcript is related to angiogenesis HSP90AB1. These hallmarks are all associated to tumor progression, which support the poor prognosis of triple negative tumors.

The luminal A sub-networks of up-regulated genes pointed to functions associated to genome instability and mutation (EIF4A3), and tumor-promoting inflammation (KPNA2). Moreover, luminal A cells overexpress transcripts related to cell cycle control, such as GRB2, EEF1G, MCM7, CSNK2B, PAK2, TK1, MAPK13, and NPM1; as well as ERBB2/3, PAK2, TK1, ICT1, and NPM1, involved in the resistance of cells to death. Concerning the genes related to tumor progression, we found HSP90AB1, GRB2, ERBB2/3, EIF4A3, HDGF, and CSNK2B. Luminal B sub-networks were similar to those of luminal A cells; this similarity is not surprising because luminal A and B cells were also grouped in only one category, i.e., luminal, as shown in S1 Table. Comparison of luminal A and B cell lines with triple negative showed up-regulation of (i) the evading growth factors ERBB2/3 in luminal A, and (ii) YWHAB and PA2G4 in luminal B. YWHAB and PA2G4 have been implicated in cell resistance to death, the sustainment of proliferative signaling and invasion and metastasis activation (see S1 Table).

To gain insight into the significance of the network circuit related to breast tumor progression, we also classified down-regulated genes with respect to functional biology and hallmarks of cancer (see S4 Table) [17,22,25,42–56]. The down-regulated genes covered a wide range of processes implicated in cancer biology. As expected, down-regulated genes are preferentially related to the maintenance of equilibrium conditions; we found functions such as: (i) cell death signaling (GABARAPL2, GABARAP, MAP1LC3A, TP53), (ii) cytoskeleton stability (ACTB, ACTG1, TUBA1A), and (iii) proliferation (TP53, GRP78, NFKBIA, GSK3B, BHLHE40).

To summarize, these results indicate the complexity of signaling through these networks and the massive consequences induced by protein hub deregulation on cross- talk between regulators of cellular events.

## Discussion

Our results have three major implications for cancer therapy (i) in helping to define a strategy to identify potential oncotargets for breast cancer treatment; (ii) in unveiling key regulatory circuits between down- and up-regulated genes responsible for the cell physiology of breast tumor progression; and (iii) in providing fast protein target identification in the context of personalized medicine that could match individual tumors types and histological subtypes. The methodology described should help to establish a quantitative relationship between putative oncotargets and a relevant therapeutic strategy. Our study also provides a framework for the identification of key players involved in breast malignancy, and may lead to new insights useful in the development of therapeutic interventions for breast cancer treatment and prevention. Further work is required to functionally validate these oncotargets starting with a pre- clinical testing at an *in vitro* level.

Although several reports have demonstrated the importance of protein networks in breast cancer [57,58], only a few studies have identified the expression profile of their corresponding genes [59,60]. While other reports have addressed protein networks subtype- specific cell lines in breast cancer, none of them has normalized expression patterns of these malignant cells to a non-tumoral breast cell line. Our report focuses on the profiling of gene expression of hub proteins, which emerge as suitable for drug development with a lower rate of negative collateral effects for patient health. Actually, as for type I and type II errors, there is a compromise concerning the choice of protein targets with a p-value of 0.1% or 5%. The number of targets available under a p-value of 0.1% is of course lower than under 5%, but, in contrast, their inhibition is expected to cause fewer side effects to the patient because of a larger difference of gene expression between malignant and normal cells. By contrast, when considering a p-value of 5%, the number of potential target increases, but the price to be pay is a higher level of adverse side effects.

The full set of interacting human proteins that we used is based on ∼10,000 genes, which is about one third of the whole human genome set that has been evaluated to be ∼30,000 based on data of expressed sequence tags [61]; a sample of one third of the whole human gene set is considered here as highly statistically significant. Breitkreutz et al. [10] showed that signaling network modeling is suitable for cancer hallmarks identification as it provides important insights into how gene mutations may affect cell physiology and lead to cancer as well as to identify putative cancer biomarkers.

The existence of an interacting sub-network between down- and up-regulated genes indicates that the differentially expressed genes, in addition to being induced by specific cancer pathways, are interacting with each other apparently in a compensatory way, which further indicates that tumorigenesis and tumoral progression require multiple and crosstalk signaling. The networks associated to different cell subtypes and their specific patterns observed here are in good agreement with the data from the literature.

With the fast pace of modern technology development, we can make a safe prediction that at some point in a not-too-distant future, when a patient is diagnosed with cancer, it will still be possible to sequence both malignant and normal cells through biopsy in order to inform the treatment plan. When specific oncotargets are identified, it will become theoretically possible to define a personalized drug cocktail on the basis of existing knowledge or even, on the fly, by *in silico* simulations (docking and molecular dynamics) of inhibitors with these oncotargets. Theoretically, this strategy is compatible with individual medicine, in the sense, that whenever the strategy is designed, it can be, in principle, largely automated. As the response rates to a specific chemotherapeutic drug might be relatively low in an unselected pre-treated patient population, it is a pre- requisite, that the repurposing strategy includes pre-selection of those patients with a favorable molecular profile in their cancer cells, i.e., those patients with the highest likelihood to benefit from the treatment. Our strategy differs from the traditional view of drug repurposing in expecting to find new indications for cocktail therapies that should affect essential pathways/mechanisms resulting in cancer cell death with minimal side effects for normal cells. In other words, we simultaneously aim to maximize efficacy and minimize toxicity of a given treatment regimen. This strategy is expected to overcome intrinsic and acquired resistance, tumor heterogeneity, adaptation, and genetic instability of cancer cells. However, multiple alternative signaling routes exist in tumors that make them resistant to drug treatment. Thus, a number of issues should be addressed to ascertain that the strategy proposed is as powerful as predicted since biological complexity always brings unexpected situations.

Importantly, the entire set of predicted drug targets has been experimentally validated by available drugs or siRNA (S5 Table) [36,38,40,62–86], which shows that the approach presented here is consistent with the state of the art and should behave similarly in new situations where knowledge is scarce. Thus, the approach described here can conceivably be implemented for a substantial number of currently used chemotherapeutic drugs, since their molecular mechanisms of action are well understood with thousands of studies available in the literature. It is hoped that our strategy will allow the elucidation of molecular networks of different tumors and histological subtypes. We believe that this strategy is valuable and can, potentially, add new tools to the armamentarium of drugs at the disposal of oncologists.

Since malignant transformation has been described as involving a defined set of physiological changes, we classified these top 5-genes into the hallmarks of cancer, according to the criteria and examples proposed by Hanahan and Weinberg [3] and based on the current functional understanding of the genes from BLAST to gene ontology (Blast2GO). GO terms are used to identify a list of potential components, functions and processes that are significantly pinpointed in the selected genes. This classification revealed that top five up- and down-regulated genes for each cell line contribute to all six acquired capabilities required for tumor progression. These results suggest that our approach is useful in identifying oncotargets suitable for breast cancer treatment. It seems reasonable to find genes involved in cell cycle, considering that the dataset concerns breast cancer and proliferating malignant cells where expression of cell cycle regulators is crucial and reflecting the high mitotic index typically associated with breast tumors. Indeed, since highly proliferating cells require energy, glycolysis is a major pathway involved in energy production. According to this landscape, our results reveal GABARAPL1 and GAPDH as hubs in BT-20 cells (triple negative). Another class of targets is the group of genes involved in cell signaling and cell communication such as membrane proteins, HER2 and 3 or EGFR, signal transduction proteins such as MYC, TK1, NPM, YWHAB, MCM7, EIF4A3, HDGF, GRB2, CHD3, PAK2, PA2G4, and transport proteins such as KPNA2. It is not surprising that in this work, we pinpointed control genes of the cell cycle or apoptosis such as MAPK13, HSP90AB1, MAGOH, CSNK2B, EEF1G, PDIA3, ICT1, SRPK1, and also those involved in the EMT process such as VIM, which play a major role in tumor development.

We identified HSP90AB1 as the only up-regulated protein hub common to all cell types in our study, which highlights the fact that the cancer subtypes addressed here all share a core of proliferative signaling pathways common in breast cancers, but with many specificities. The part of the expression pattern that is common to all cell lines included can further be influenced by the underlying genetic background of the tumor cells and the stage of tumor progression at which the cell line was derived.

To further demonstrate the predictive power of subtype-specific networks, we attempted to predict subtype-specific therapeutic interventions. If a hub gene specifically appears in either a luminal A, B or triple-negative subtype-specific network, we expect that this gene could be a drug target specific for this subtype. Based on this criterion, GRB2, ICT1, PDIA3, KPNA2, NPM, PAK2, EIF4A3 and MCM7 are predicted as potential drug targets specific to the luminal A cell type, PA2G4 would be specific to the luminal B cell type, and MAGOH, MYC, SRPK1, VIM, GABARAPL1, GAPDH and CHD3 are predicted as potential drug targets specific of the triple negative subtype.

We also found associations between the down-regulation of genes and therapy, which in some instances provide insights into the interplay between tumor suppressors and the cellular machinery in mediating drug sensitivity. For example, Zheng et al. [87] showed that overexpression of HER-2/*neu* could decrease the amount of wild- type p53 protein via the activation of the PI3K pathway, and the induction of MDM2 nuclear translocation in MCF-7 human breast cancer cells. Blockage of the PI3K pathway with its specific inhibitor LY294002 caused G1-S phase arrest, decreased the cell growth rate and increased chemo- and radio-therapeutic sensitivity in MCF-7 cells expressing wild-type p53. Additionally, Wang et al. [88] showed that abrogation of GRP78 induced sensitivity of breast cancer cells to taxol and vinblastine.

## Conclusions

By using an integrative network analysis of the data derived from transcriptome and interactome public resources, we have predicted selective combinations of druggable targets to control key pathways in breast cancer. The molecular alterations observed in breast cancer cell lines represent either driver events and/or driver pathways that are necessary for breast cancer development or progression. However, it is clear that signaling mechanisms of the luminal A, B and triple negative subtypes are different. Furthermore, the up- and down-regulated networks predicted subtype-specific drug targets and possible compensation circuits between up- and down-regulated genes. Together with the finding that more connected genes could act as cancer regulators, these results may have significant clinical implications in the personalized treatment of cancer patients since every breast cancer can be considered as unique and reflects distinct qualitative and quantitative molecular traits. Thus, the knowledge of the entire set of molecular traits carried by any given breast cancer and patient is required for actual personalized therapy to be realized.

## Methods

### Interactome data

The protein connectivity inferences described below are based on the protein interactions given in the file intact-micluster.zip available from ftp://ftp.ebi.ac.uk/pub/databases/intact/current/psimitab/ (accessed on 04.04.2014). We selected the two columns of UniprotKB identifiers (UID) in the intact-micluster.zip file and eliminated the incomplete pairs (marked as “-”, i.e., when an intact access number has no UniprotKB equivalent known). The resulting file contained 308,314 protein pairs. This interaction file was then processed to form a non-redundant UID list used to retrieve the corresponding protein sequences (68,504) by querying UniprotKB at http://www.uniprot.org/help/uniprotkb. Since some UID were obsolete, we substituted them by their current name retrieved by querying the field *search* at UniprotKB using the format ‘replaces:*obsolete UID’*. The equivalence between UID and human genes was obtained by homology search (tBLASTn) of protein sequences (68,504) used as queries and human coding sequences (CDS) used as subjects from the dataset (hs37p1.EID.tar.gz) of Fedorov’s laboratory [89] available at http://bpg.utoledo.edu/∼afedorov/lab/eid.html. Homologous hits were consideredsignificant when their score was ≥120, E-value ≤10^−4^ and identity rate ≥80% over ≥50%of query size (http://mitointeractome.kobic.kr/supplement.php). After elimination of subject redundancy (keeping the hit matching the largest identity rate), the final size of human CDS dataset fully described by protein interactions was 17,301.

### Transcriptome data

We recovered transcriptome datasets of cell lines (BT-20, BT-474, MDA-MB-231, MDA-MB-468, MCF-7, MCF10A, T-47D, ZR-75-1, see information at http://www.atcc.org/) from http://www.illumina.com/science/data_library.ilmn. The gene expression profile was evaluated through a homology search with the human CDS sample of the Fedorov’s laboratory. The fifty bp sequences from transcriptome tags were used as queries in homology searches (BLASTn) in human CDSs. The homology redundancy in the BLASTn output file gave us the tag count per gene, i.e., a profile of human gene expression for the considered sample. Homologous hits were considered significant when covering ≥25 bp (50% of size).

Each gene expression profile (tag count per gene) was normalized according to CDS size and whole tag count using the formula (10^9^*C)/(N*L), where 10^9^ is a correction factor, *C* is the number of reads that match a gene, *N* is the total mappable tags in the experiment, and *L* is the CDS size [90]. When tags were counted for more than one gene isoform (alternative splicing forms), we cumulated counts and allocated them to just one form (the largest one); this strategy means that we looked for gene expression and not isoform expression.

To allow the comparison between independent gene expression profiles, we further applied Quantil-normalization (*Q*-norm) considering the eight samples of this study [91]. Up- and down-regulated genes were obtained by subtracting expression values (pair-wise comparisons) of a file containing malignant cell data from a control file (data from a non-tumoral breast cell line) and sorting on differential expression values (negative and positive values for down- and up-regulated genes, respectively). Then, we searched the best fit (95%) performed with a Gaussian function using PRISM (http://www.graphpad.com/scientific-software/prism/) on data of log_10_(x_i_+1) of the difference of expression values, where *x*_i_ represents the values obtained from the subtraction of the two transcriptome data under comparison. The best fit is a necessary step to find the classification thresholds corresponding to *p*-values 0.05 and 0.001 from a theoretical distribution.

Lists gathering only significantly down- and up-regulated genes were prepared and compared to the list of interaction pairs (see previous section). Non-connected nodes were eliminated from this list by recursive filtering, first, on the subgroup of edges and, second, on the query list of nodes itself. The final list of interacting nodes with their respective expression values and connectivity was analyzed graphically using GEPHI (http://gephi.github.io/).

### Classification of genes according to expression rate

Because genes with a low expression rate are the most numerous, the distribution of gene frequency according to normalized tag counts has the shape of a Poisson distribution. To classify genes into down- and up-regulated, a symmetrical distribution is necessary in order to estimate a *p*-value on a Gaussian distribution resulting from the best fit with the observed distribution.

To obtain a symmetrical distribution, we subtracted the normalized (according to size and number) data from the transcriptome of a malignant cell line from that of the non-tumoral cell line (MCF10A). After normalization using *Q*-norm this distribution had a mean close to zero for any comparison between a malignant cell line and the control. The log_10_(*x*_i_+1) had the effect to bring the observed distribution closer to a Gaussian distribution. We used PRISM to perform the best fit (95%) with a Gaussian distribution of log_10_(*x*_i_+1) data classified by increasing values from the larger negative number to the largest positive number. The boundaries corresponding to *p*-values of 5% and 1‰ considering a two-tails *p*-value (2.5% or 0.05% on both sides) on the best fit of a Gaussian distribution were used to calculate the classification threshold of down- and up-regulated genes on the observed distribution using the inverse function, i.e., 10^log_10_(*x*i+1)^ and subtracting 1 from the result of the exponential. The down-regulated genes at *p*-values <0.05 and <0.001 were those with negative values lower than classification thresholds of −90 and −150, respectively, and the up- regulated genes at *p*- values <0.05 and <0.001 were those with positive values higher than classification thresholds of +80 and +90, respectively, according to the case under consideration.

### Connectivity

The connection rate (relative number of edges per node) or connectivity of a protein with the others is used here in reference to the work of Breitkreutz et al. [10] because protein hubs were shown to be putative targets suitable for drug development. Protein connectivity has been calculated (by counting the connection pairs per node) on the full set of interacting proteins in this investigation as well as on samples of down- and up-regulated genes. *Betweenness centrality* was also taken into account using GEPHI. The connectivity is given, here, relative to the sum of connection for the network under consideration.

Since Breitkreutz et al. [10] considered the proteins with the largest rate of betweenness centrality as the most relevant targets for drug development; we searched and analyzed the top-5 most connected proteins.

### Graphical network representation using Gephi

Networks and sub-networks were analyzed graphically in the GEPHI environment by pasting data in the input node file and by using its toolbox to calculate and represent protein connectivity as well as betweenness centrality automatically.

### Functional annotation of genes

In order to annotate up-regulated genes, we searched for homologies among the sequences corresponding to the sub-networks against *nr* (GenBank, rel 181) using the BLAST to gene ontology—Blast2GO [92].

## Supporting Information

S1 Table Up-regulated genes with top-5 connectivity in malignant cell lines of breast compared to a normal cell line (MCF-10A) used as a control. (DOC)

S2 Table Down-regulated genes with top-5 connectivity in malignant cell lines of breast compared to a normal cell line (MCF-10A) used as a control. (DOC)

S3 Table Top-5 up-regulated genes and GO classification. (DOC)

S4 Table Top-5 down-regulated genes and GO classification. (DOC)

S5 Table Therapeutic and/or *in vitro* compounds that show significant specificity in treatment. (DOC)
