# Supplementary material for: A Computational Strategy to Select Optimized Protein Targets for Drug Development toward the Control of Cancer Diseases
Source: PLoS One. 2015 Jan 27;10(1):e0115054. doi: 10.1371/journal.pone.0115054 (PMC4308075; doi:10.1371/journal.pone.0115054)
Supplement: S1 Table — (DOC) [file pone.0115054.s001.doc]

**Table S1**. Up-regulated genes with top-5 connectivity in malignant cell lines of breast compared to a normal cell line (MCF-10A) used as a control.

|  |  | **Uniprotkb** | **Protein Name** | **Gene Name** |
| --- | --- | --- | --- | --- |
|  |  | **Triple-Negative** | |  |
|  |  | *MDA-MB-468 (95%)* | |  |
|  |  | P00533 | Epidermal growth factor receptor | EGFR |
|  |  | P08238 | Heat shock protein HSP 90-beta | HSP90AB1 |
|  |  | O15264 | Mitogen-activated protein kinase 13 | MAPK13 |
|  |  | P31946 | 14-3-3 protein beta/alpha | YWHAB |
|  |  | P61326 | Protein mago nashi homolog | MAGOH |
|  |  | *MDA-MB-468 (100%)* | |  |
|  |  | P00533 | Epidermal growth factor receptor | EGFR |
|  |  | P08238 | Heat shock protein HSP 90-beta | HSP90AB1 |
|  |  | P61326 | Protein mago nashi homolog | MAGOH |
|  |  | P26641 | Elongation factor 1-gamma | EEF1G |
|  |  | P67870 | Casein kinase II subunit beta | CSNK2B |
|  |  | *MDA-MB-231 (95%)* | |  |
|  |  | P01106 | Myc proto-oncogene protein | MYC |
|  |  | P08238 | Heat shock protein HSP 90-beta | HSP90AB1 |
|  |  | O15264 | Mitogen-activated protein kinase 13 | MAPK13 |
|  |  | P31946 | 14-3-3 protein beta/alpha | YWHAB |
|  |  | Q96SB4 | SRSF protein kinase 1 | SRPK1 |
|  |  | MDA-MB-231 (100%) | |  |
|  |  | P08238 | Heat shock protein HSP 90-beta | HSP90AB1 |
|  |  | P08670 | Vimentin | VIM |
|  |  | P67870 | Casein kinase II subunit beta | CSNK2B |
|  |  | P31946 | 14-3-3 protein beta/alpha | YWHAB |
|  |  | P04183 | Thymidine kinase, cytosolic | TK1 |
|  |  | *BT-20 (95%)* | |  |
|  |  | Q9H0R8 | Gamma-aminobutyric acid receptor-associated protein-like 1 | GABARAPL1 |
|  |  | P00533 | Epidermal growth factor receptor | EGFR |
|  |  | P08238 | Heat shock protein HSP 90-beta | HSP90AB1 |
|  |  | P67870 | Casein kinase II subunit beta | CSNK2B |
|  |  | P04406 | Glyceraldehyde-3-phosphate dehydrogenase | GAPDH |
|  |  | *BT-20 (100%)* | |  |
|  |  | P00533 | Epidermal growth factor receptor | EGFR |
|  |  | P08238 | Heat shock protein HSP 90-beta | HSP90AB1 |
|  |  | Q12873 | Chromodomain-helicase-DNA-binding protein 3 | CHD3 |
|  |  | P04406 | Glyceraldehyde-3-phosphate dehydrogenase | GAPDH |
|  |  | P51858 | Hepatoma-derived growth factor | HDGF |
|  |  | ***Luminal A***  *MCF-7 (95%)* | |  |
|  |  | P62993 | Growth factor receptor-bound protein 2 | GRB2 |
|  |  | P08238 | Heat shock protein HSP 90-beta | HSP90AB1 |
|  |  | P21860 | Receptor tyrosine-protein kinase erbB-3 | ERBB3 |
|  |  | P38919 | Eukaryotic initiation factor 4A-III | EIF4A3 |
|  |  | P26641 | Elongation factor 1-gamma | EEF1G |
|  |  | *MCF-7 (100%)* | |  |
|  |  | P62993 | Growth factor receptor-bound protein 2 | GRB2 |
|  |  | P08238 | Heat shock protein HSP 90-beta | HSP90AB1 |
|  |  | P26641 | Elongation factor 1-gamma | EEF1G |
|  |  | P33993 | DNA replication licensing factor MCM7 | MCM7 |
|  |  | P52292 | Importin subunit alpha-1 | KPNA2 |
|  |  | *T-47D (95%)* | |  |
|  |  | P62993 | Growth factor receptor-bound protein 2 | GRB2 |
|  |  | P08238 | Heat shock protein HSP 90-beta | HSP90AB1 |
|  |  | P21860 | Receptor tyrosine-protein kinase erbB-3 | ERBB3 |
|  |  | P51858 | Hepatoma-derived growth factor | HDGF |
|  |  | Q13177 | Serine/threonine-protein kinase PAK 2 | PAK2 |
|  |  | *T-47D (100%)* | |  |
|  |  | P08238 | Heat shock protein HSP 90-beta | HSP90AB1 |
|  |  | P21860 | Receptor tyrosine-protein kinase erbB-3 | ERBB3 |
|  |  | Q13177 | Serine/threonine-protein kinase PAK 2 | PAK2 |
|  |  | P04183 | Thymidine kinase, cytosolic | TK1 |
|  |  | P67870 | Casein kinase II subunit beta | CSNK2B |
|  |  | *ZR-75-1 (95%)* | |  |
|  |  | P62993 | Growth factor receptor-bound protein 2 | GRB2 |
|  |  | P08238 | Heat shock protein HSP 90-beta | HSP90AB1 |
|  |  | Q14197 | Peptidyl-tRNA hydrolase ICT1, mitochondrial | ICT1 |
|  |  | P04626 | Receptor tyrosine-protein kinase erbB-2 | ERBB2 |
|  |  | O15264 | Mitogen-activated protein kinase 13 | MAPK13 |
|  |  | *ZR-75-1 (100%)* | |  |
|  |  | P62993 | Growth factor receptor-bound protein 2 | GRB2 |
|  |  | P08238 | Heat shock protein HSP 90-beta | HSP90AB1 |
|  |  | P30101 | Protein disulfide-isomerase A3 | PDIA3 |
|  |  | P52292 | Importin subunit alpha-1 | KPNA2 |
|  |  | P06748 | Nucleophosmin | NPM1 |
|  |  | ***Luminal B***  *BT-474 (95%)* | |  |
|  |  | P08238 | Heat shock protein HSP 90-beta | HSP90AB1 |
|  |  | P04626 | Receptor tyrosine-protein kinase erbB-2 | ERBB2 |
|  |  | P21860 | Receptor tyrosine-protein kinase erbB-3 | ERBB3 |
|  |  | P31946 | 14-3-3 protein beta/alpha | YWHAB |
|  |  | P67870 | Casein kinase II subunit beta | CSNK2B |
|  |  | *BT-474 (100%)* | |  |
|  |  | P08238 | Heat shock protein HSP 90-beta | HSP90AB1 |
|  |  | P04626 | Receptor tyrosine-protein kinase erbB-2 | ERBB2 |
|  |  | P21860 | Receptor tyrosine-protein kinase erbB-3 | ERBB3 |
|  |  | P31946 | 14-3-3 protein beta/alpha | YWHAB |
|  |  | Q9UQ80 | Proliferation-associated protein 2G4 | PA2G4 |

. Down-regulated genes with top-5 connectivity in malignant cell lines of breast compared to a normal cell line (MCF-10A) used as a control.

|  |  |  | |  | | | | |
| --- | --- | --- | --- | --- | --- | --- | --- | --- |
|  | | | |  | | | | |
|  | | | |  | | | | |
|  |  |  | |  | | | | |
|  |  |  | |  | | | | |
|  |  |  | |  | | | | |
|  |  |  | |  | | | | |
|  |  |  | |  | | | | |
|  | | | |  | | | | |
|  |  |  | |  | | | | |
|  |  |  | |  | | | | |
|  |  |  | |  | | | | |
|  |  |  | |  | | | | |
|  |  |  | |  | | | | |
|  | | | |  | | | | |
|  |  |  | |  | | | | |
|  |  |  | |  | | | | |
|  |  |  | |  | | | | |
|  |  |  | |  | | | | |
|  |  |  | |  | | | | |
|  | | | |  | | | | |
|  |  |  | |  | | | | |
|  |  |  | |  | | | | |
|  |  |  | |  | | | | |
|  |  |  | |  | | | | |
|  |  |  | |  | | | | |
|  | | | |  | | | | |
|  |  |  | |  | | | | |
|  |  |  | |  | | | | |
|  |  |  | |  | | | | |
|  |  |  | |  | | | | |
|  |  |  | |  | | | | |
|  | |  | |  | |  | | |
|  |  |  | |  | | | | |
|  |  |  | |  | | | | |
|  |  |  | |  | | | | |
|  |  |  | |  | | | | |
|  |  |  | |  | | | | |
|  | | | |  | | | | |
|  | |  |  |  | | | | |
|  |  |  | |  | | | | |
|  |  |  | |  | | | | |
|  |  |  | |  | | | | |
|  |  |  | |  | | | | |
|  |  |  | |  | | | | |
|  | |  | |  |  | | | |
|  |  |  | |  | | | | |
|  |  |  | |  | | | | |
|  |  |  | |  | | | | |
|  |  |  | |  | | | | |
|  |  |  | |  | | | | |
|  | |  | |  | | | |  |
|  |  |  | |  | | | | |
|  |  |  | |  | | | | |
|  |  |  | |  | | | | |
|  |  |  | |  | | | | |
|  |  |  | |  | | | | |
|  | |  | |  | |  | | |
|  |  |  | |  | | | | |
|  |  |  | |  | | | | |
|  |  |  | |  | | | | |
|  |  |  | |  | | | | |
|  |  |  | |  | | | | |
|  | | | |  | | | | |
|  |  |  | |  | | | | |
|  |  |  | |  | | | | |
|  |  |  | |  | | | | |
|  |  |  | |  | | | | |
|  |  |  | |  | | | | |
|  | | | |  | | | | |
|  |  |  | |  | | | | |
|  |  |  | |  | | | | |
|  |  |  | |  | | | | |
|  |  |  | |  | | | | |
|  |  |  | |  | | | | |
|  | |  | |  | | |  | |
|  | | | |  | | | | |
|  |  |  | |  | | | | |
|  |  |  | |  | | | | |
|  |  |  | |  | | | | |
|  |  |  | |  | | | | |
|  |  |  | |  | | | | |
|  | | | |  | | | | |
|  |  |  | |  | | | | |
|  |  |  | |  | | | | |
|  |  |  | |  | | | | |
|  |  |  | |  | | | | |
|  |  |  | |  | | | | |

. Top-5 up-regulated genes and GO classification.

|  |  |  |  |  |  |
| --- | --- | --- | --- | --- | --- |
|  |  |  |  |  |  |
|  |  |  |  |  |  |
|  |  |  |  |  |  |
|  |  |  |  |  |  |
|  |  |  |  |  |  |
|  |  |  |  |  |  |
|  |  |  |  |  |  |
|  |  |  |  |  |  |
|  |  |  |  |  |  |
|  |  |  |  |  |  |
|  |  |  |  |  |  |
|  |  |  |  |  |  |
|  |  |  |  |  |  |
|  |  |  |  |  |  |
|  |  |  |  |  |  |
|  |  |  |  |  |  |
|  |  |  |  |  |  |
|  |  |  |  |  |  |
|  |  |  |  |  |  |
|  |  |  |  |  |  |
|  |  |  |  |  |  |
|  |  |  |  |  |  |
|  |  |  |  |  |  |
|  |  |  |  |  |  |
|  |  |  |  |  |  |
|  |  |  |  |  |  |

. Top-5 down-regulated genes and GO classification

|  |  |  |  |  |  |
| --- | --- | --- | --- | --- | --- |
|  |  |  |  |  |  |
|  |  |  |  |  |  |
|  |  |  |  |  |  |
|  |  |  |  |  |  |
|  |  |  |  |  |  |
|  |  |  |  |  |  |
|  |  |  |  |  |  |
|  |  |  |  |  |  |
|  |  |  |  |  |  |
|  |  |  |  |  |  |
|  |  |  |  |  |  |
|  |  |  |  |  |  |
|  |  |  |  |  |  |
|  |  |  |  |  |  |
|  |  |  |  |  |  |
|  |  |  |  |  |  |
|  |  |  |  |  |  |
|  |  |  |  |  |  |

compounds that show significant specificity in treatment

|  |  |  |  |
| --- | --- | --- | --- |
|  |  |  |  |
|  |  |  |  |
|  |  |  |  |
|  |  |  |  |
|  |  |  |  |
|  |  |  |  |
|  |  |  |  |
|  |  |  |  |
|  |  |  |  |
|  |  |  |  |
|  |  |  |  |
|  |  |  |  |
|  |  |  |  |
|  |  |  |  |
|  |  |  |  |
|  |  |  |  |
|  |  |  |  |
|  |  |  |  |
|  |  |  |  |
|  |  |  |  |
|  |  |  |  |
|  |  |  |  |
|  |  |  |  |
|  |  |  |  |
|  |  |  |  |
|  |  |  |  |
