# Supplementary material for: A Computational Strategy to Select Optimized Protein Targets for Drug Development toward the Control of Cancer Diseases
Source: PLoS One. 2015 Jan 27;10(1):e0115054. doi: 10.1371/journal.pone.0115054 (PMC4308075; doi:10.1371/journal.pone.0115054)
Supplement: S2 Table — (DOC) [file pone.0115054.s002.doc]

**Table S2**. Down-regulated genes with top-5 connectivity in malignant cell lines of breast compared to a normal cell line (MCF-10A) used as a control.

| **UniProtkb** | **Protein Name** | **Gene Name** | |  | | | | |
| --- | --- | --- | --- | --- | --- | --- | --- | --- |
| ***Triple-Negative*** | | | |  | | | | |
| *MDA-MB-468 (95%)* | | | |  | | | | |
| P60520 | Gamma-aminobutyric acid receptor-associated protein-like 2 | GABARAPL2 | |  | | | | |
| O95166 | Gamma-aminobutyric acid receptor-associated protein | GABARAP | |  | | | | |
| Q9H492 | Microtubule-associated proteins 1A/1B light chain 3A | MAP1LC3A | |  | | | | |
| P60709 | Actin, cytoplasmic 1 | ACTB | |  | | | | |
| P08670 | Vimentin | VIM | |  | | | | |
| *MDA-MB-468 (100%)* | | | |  | | | | |
| O95166 | Gamma-aminobutyric acid receptor-associated protein | GABARAP | |  | | | | |
| P60709 | Actin, cytoplasmic 1 | ACTB | |  | | | | |
| P08670 | Vimentin | VIM | |  | | | | |
| P11021 | 78 kDa glucose-regulated protein | HSPA5 | |  | | | | |
| P25963 | NF-kappa-B inhibitor alpha | NFKBIA | |  | | | | |
| *MDA-MB-231 (95%)* | | | |  | | | | |
| Q9H492 | Microtubule-associated proteins 1A/1B light chain 3A | MAP1LC3A | |  | | | | |
| P60709 | Actin, cytoplasmic 1 | ACTB | |  | | | | |
| P49841 | Glycogen synthase kinase-3 beta | GSK3B | |  | | | | |
| O43889 | Cyclic AMP-responsive element-binding protein 3 | CREB3 | |  | | | | |
| P25963 | NF-kappa-B inhibitor alpha | NFKBIA | |  | | | | |
| *MDA-MB-231 (100%)* | | | |  | | | | |
| P60709 | Actin, cytoplasmic 1 | ACTB | |  | | | | |
| P25963 | NF-kappa-B inhibitor alpha | NFKBIA | |  | | | | |
| Q99959 | Plakophilin-2 | PKP2 | |  | | | | |
| Q71U36 | Tubulin alpha-1A chain | TUBA1A | |  | | | | |
| O14503 | Class E basic helix-loop-helix protein 40 | BHLHE40 | |  | | | | |
| *BT-20 (95%)* | | | |  | | | | |
| O95166 | Gamma-aminobutyric acid receptor-associated protein | GABARAP | |  | | | | |
| Q9H492 | Microtubule-associated proteins 1A/1B light chain 3A | MAP1LC3A | |  | | | | |
| P60709 | Actin, cytoplasmic 1 | ACTB | |  | | | | |
| P08670 | Vimentin | VIM | |  | | | | |
| P11021 | 78 kDa glucose-regulated protein | HSPA5 | |  | | | | |
| *BT-20 (100%)* | |  | |  | |  | | |
| P60709 | Actin, cytoplasmic 1 | ACTB | |  | | | | |
| P08670 | Vimentin | VIM | |  | | | | |
| P25963 | NF-kappa-B inhibitor alpha | NFKBIA | |  | | | | |
| P63261 | Actin, cytoplasmic 2 | ACTG1 | |  | | | | |
| P04083 | Annexin A1 | ANXA1 | |  | | | | |
| ***Luminal A*** | | | |  | | | | |
| *MCF-7 (95%)* | |  |  |  | | | | |
| P60520 | Gamma-aminobutyric acid receptor-associated protein-like 2 | GABARAPL2 | |  | | | | |
| O95166 | Gamma-aminobutyric acid receptor-associated protein | GABARAP | |  | | | | |
| Q9H492 | Microtubule-associated proteins 1A/1B light chain 3A | MAP1LC3A | |  | | | | |
| P60709 | Actin, cytoplasmic 1 | ACTB | |  | | | | |
| P49841 | Glycogen synthase kinase-3 beta | GSK3B | |  | | | | |
| *MCF-7 (100%)* | |  | |  |  | | | |
| P60520 | Gamma-aminobutyric acid receptor-associated protein-like 2 | GABARAPL2 | |  | | | | |
| P60709 | Actin, cytoplasmic 1 | ACTB | |  | | | | |
| P08670 | Vimentin | VIM | |  | | | | |
| P25963 | NF-kappa-B inhibitor alpha | NFKBIA | |  | | | | |
| P60953 | Cell division control protein 42 homolog | CDC42 | |  | | | | |
| *T-47D (95%)* | |  | |  | | | |  |
| Q9H492 | Microtubule-associated proteins 1A/1B light chain 3A | MAP1LC3A | |  | | | | |
| P60709 | Actin, cytoplasmic 1 | ACTB | |  | | | | |
| P04637 | Cellular tumor antigen p53 | TP53 | |  | | | | |
| P31946 | 14-3-3 protein beta/alpha | YWHAB | |  | | | | |
| P08670 | Vimentin | VIM | |  | | | | |
| *T-47D (100%)* | |  | |  | |  | | |
| P60709 | Actin, cytoplasmic 1 | ACTB | |  | | | | |
| P04637 | Cellular tumor antigen p53 | TP53 | |  | | | | |
| P08670 | Vimentin | VIM | |  | | | | |
| P31946 | 14-3-3 protein beta/alpha | YWHAB | |  | | | | |
| P60953 | Cell division control protein 42 homolog | CDC42 | |  | | | | |
| *ZR-75-1 (95%)* | | | |  | | | | |
| P60520 | Gamma-aminobutyric acid receptor-associated protein-like 2 | GABARAPL2 | |  | | | | |
| O95166 | Gamma-aminobutyric acid receptor-associated protein | GABARAP | |  | | | | |
| Q9H492 | Microtubule-associated proteins 1A/1B light chain 3A | MAP1LC3A | |  | | | | |
| P60709 | Actin, cytoplasmic 1 | ACTB | |  | | | | |
| P04637 | Cellular tumor antigen p53 | TP53 | |  | | | | |
| *ZR-75-1 (95%)* | | | |  | | | | |
| P60709 | Actin, cytoplasmic 1 | ACTB | |  | | | | |
| P31946 | 14-3-3 protein beta/alpha | YWHAB | |  | | | | |
| P08670 | Vimentin | VIM | |  | | | | |
| P25963 | NF-kappa-B inhibitor alpha | NFKBIA | |  | | | | |
| P63261 | Actin, cytoplasmic 2 | ACTG1 | |  | | | | |
| ***Luminal B*** | |  | |  | | |  | |
| *BT-474 (95%)* | | | |  | | | | |
| P60520 | Gamma-aminobutyric acid receptor-associated protein-like 2 | GABARAPL2 | |  | | | | |
| O95166 | Gamma-aminobutyric acid receptor-associated protein | GABARAP | |  | | | | |
| P60709 | Actin, cytoplasmic 1 | ACTB | |  | | | | |
| P08670 | Vimentin | VIM | |  | | | | |
| P11021 | 78 kDa glucose-regulated protein | HSPA5 | |  | | | | |
| *BT-474 (100%)* | | | |  | | | | |
| P60709 | Actin, cytoplasmic 1 | ACTB | |  | | | | |
| P08670 | Vimentin | VIM | |  | | | | |
| P11021 | 78 kDa glucose-regulated protein | HSPA5 | |  | | | | |
| P04406 | Glyceraldehyde-3-phosphate dehydrogenase | GAPDH | |  | | | | |
| P25963 | NF-kappa-B inhibitor alpha | NFKBIA | |  | | | | |
