# Supplementary material for: A Computational Strategy to Select Optimized Protein Targets for Drug Development toward the Control of Cancer Diseases
Source: PLoS One. 2015 Jan 27;10(1):e0115054. doi: 10.1371/journal.pone.0115054 (PMC4308075; doi:10.1371/journal.pone.0115054)
Supplement: S3 Table — (DOC) [file pone.0115054.s003.doc]

**Table S3**. Top-5 up-regulated genes and GO classification.

| **Uniprotkb** | **Gene Name** | **Biological Process** | **Molecular Function** | **Cellular Component** | **Hallmarks of cancer** |
| --- | --- | --- | --- | --- | --- |
| O15264 | MAPK13 | DNA replication, positive regulation of G1/S transition of mitotic cell cycle | [Kinase](http://www.uniprot.org/keywords/KW-0418), [serine/threonine-protein kinase](http://www.uniprot.org/keywords/KW-0723), [transferase](http://www.uniprot.org/keywords/KW-0808) | Clathrin coat of endocytic vesicle, plasma membrane, centrosome, spindle pole, cell junction | Sustaining proliferative signaling [3] |
| P00533 | EGFR | Phosphatidylinositol-mediated signaling, protein phosphorylation, protein oligomerization, response to DNA damage stimulus | Protein kinase activator activity, DNA helicase activity | Chromosomal, protein complex, cytoskeletal, endosomal part, organelle membrane | Sustaining proliferative signaling [13] |
| P01106 | MYC | Regulation of transcription from RNA polymerase II promoter, DNA-dependent, epithelial cell proliferation, phosphorylation, signal transduction, response to wounding, microtubule-based process, chromatin organization | Protein kinase activity, transition metal ion binding, transmembrane receptor protein kinase activity | Extracellular region, nucleoplasm, intracellular organelle part, chromosome | Sustaining proliferative signaling, resisting cell death, deregulating cellular energetics [3,14,15] |
| P04183 | TK1 | Response to chemical stimulus, small GTPase mediated signal transduction, apoptotic process, signal transduction, mitotic cell cycle phase transition | Protein and cation binding, signal transducer activity | Plasma membrane, cytoplasmic membrane-bounded vesicle | Sustaining proliferative signaling, resisting cell death [16] |
| P04406 | GAPDH | Regulation of cell proliferation, viral process, modification by symbiont of host morphology or physiology, alpha-amino acid metabolic process, inositol lipid-mediated signaling, MAPK cascade | Kinase binding, protein dimerization activity | Cytoskeleton | Deregulating cellular energetics [17] |
| P04626 | ERBB2 | Intracellular protein kinase cascade, ERBB signaling pathway, cellular response to FGF stimulus, axonogenesis, neuron projection guidance, protein localization, cellular macromolecule localization | Nucleic acid binding | Plasma membrane, protein complex | Evading growth factor, activating invasion and metastasis, resisting cell death, inducing angiogenesis [18] |
| P06748 | NPM1 | Transmembrane receptor protein tyrosine kinase signaling pathway, neurotrophin signaling pathway, regulation of apoptotic process, immune response-regulating cell surface receptor signaling pathway, positive regulation of protein phosphorylation | Protein binding, phosphotransferase activity, alcohol group as acceptor, kinase activity | Membrane-bounded organelle | Sustaining proliferative signaling, resisting cell death [19,20] |
| P08238 | HSP90AB1 | Translation, regulation of cellular macromolecule biosynthetic and protein metabolic process, posttranscriptional regulation of gene expression, cell proliferation | [Chaperone](http://www.uniprot.org/keywords/KW-0143), ATP binding | [Cytoplasm](http://www.uniprot.org/keywords/KW-0963) | Inducing  angiogenesis [21] |
| P08670 | VIM | Cell surface receptor signaling pathway, defense response, immune response, regulation of gene expression | Protein binding, transferase activity, transferring phosphorus-containing groups | Nuclear lumen, nuclear, vesicle | Activating invasion and metastasis, EMT process [22] |
| P21860 | ERBB3 | Signal transduction, gene expression, cellular macromolecule biosynthetic process, RNA biosynthetic process | Binding | Membrane | Evading growth factor, activating invasion and metastasis, resisting cell death, inducing angiogenesis [18] |
| P26641 | EEF1G | Programmed cell death, phosphorylation, protein modification process, developmental process, regulation of molecular function | Ion and organic cyclic compound binding, nucleoside phosphate binding, small molecule binding | Intracellular organelle, non-membrane-bounded organelle | Sustaining proliferative signaling [23] |
| P30101 | PDIA3 | Developmental process, phosphate-containing compound metabolic process, macromolecule and nucleobase-containing compound metabolic process | Binding | [Endoplasmic reticulum](http://www.uniprot.org/locations/SL-0095) | Resisting cell death [24] |
| P31946 | YWHAB | Regulation of cellular process, cellular response to chemical stimulus, response to organic substance | Binding | Intracellular organelle part, nucleus | Sustaining proliferative signaling, resisting cell death, activating invasion and metastasis [25] |
| P33993 | MCM7 | Macromolecule metabolic process, response to stimulus, regulation of cellular process, gene expression, protein modification process | [Helicase](http://www.uniprot.org/keywords/KW-0347), [hydrolase](http://www.uniprot.org/keywords/KW-0378) | Cytoplasmic | Sustaining proliferative signaling [26] |
| P38919 | EIF4A3 | [mRNA processing](http://www.uniprot.org/keywords/KW-0507), [mRNA splicing](http://www.uniprot.org/keywords/KW-0508), [mRNA transport](http://www.uniprot.org/keywords/KW-0509), [nonsense-mediated mRNA decay](http://www.uniprot.org/keywords/KW-0866), [rRNA processing](http://www.uniprot.org/keywords/KW-0698), [translation regulation](http://www.uniprot.org/keywords/KW-0810), [transport](http://www.uniprot.org/keywords/KW-0813) | [Helicase](http://www.uniprot.org/keywords/KW-0347), [hydrolase](http://www.uniprot.org/keywords/KW-0378) | [Cytoplasm](http://www.uniprot.org/keywords/KW-0963), [nucleus](http://www.uniprot.org/keywords/KW-0539), [spliceosome](http://www.uniprot.org/keywords/KW-0747) | Genome instability and mutation [27] |
| P51858 | HDGF | Single organism signaling, cell communication, regulation of cellular process, cellular response to stimulus | [Growth factor](http://www.uniprot.org/keywords/KW-0339), [repressor](http://www.uniprot.org/keywords/KW-0678) | [Cytoplasm](http://www.uniprot.org/locations/SL-0086), [nucleus](http://www.uniprot.org/locations/SL-0191) | Activating invasion and metastasis, EMT process; resisting cell death [28,29] |
| P52292 | KPNA2 | Single organism signaling, cell communication, regulation of cellular process, cellular response to stimulus, transport, cellular nitrogen compound metabolic process, | Binding | Membrane-enclosed lumen, intracellular organelle lumen, nuclear | Tumor promoting inflammation [30] |
| P61326 | MAGOH | Cellular biosynthetic process, regulation of biosynthetic process, regulation of cellular metabolic process | [Poly(A) RNA binding](http://www.ebi.ac.uk/QuickGO/GTerm?id=GO:0044822), [protein binding](http://www.ebi.ac.uk/QuickGO/GTerm?id=GO:0005515) | Intracellular membrane-bounded organelle | Sustaining proliferative signaling [31] |
| P62993 | GRB2 | Response to stimulus, macromolecule biosynthetic process | Binding | [Cytoplasm](http://www.uniprot.org/keywords/KW-0963), [endosome](http://www.uniprot.org/keywords/KW-0967), [golgi apparatus](http://www.uniprot.org/keywords/KW-0333), [nucleus](http://www.uniprot.org/keywords/KW-0539) | Sustaining proliferative signaling; activating invasion and metastasis [32] |
| P67870 | CSNK2B | Protein metabolic process, cellular macromolecule metabolic process | [Protein binding](http://www.ebi.ac.uk/QuickGO/GTerm?id=GO:0005515),  [protein kinase regulator activity](http://www.ebi.ac.uk/QuickGO/GTerm?id=GO:0019887),  [receptor binding](http://www.ebi.ac.uk/QuickGO/GTerm?id=GO:0005102), [transcription factor binding](http://www.ebi.ac.uk/QuickGO/GTerm?id=GO:0008134) | [Cytoplasm](http://www.ebi.ac.uk/QuickGO/GTerm?id=GO:0005737), nucleus | Sustaining proliferative signaling; activating invasion and metastasis, EMT process [33,34] |
| Q12873 | CHD3 | Regulation of metabolic process, regulation of cellular process | [Chromatin regulator](http://www.uniprot.org/keywords/KW-0156), [helicase](http://www.uniprot.org/keywords/KW-0347), [hydrolase](http://www.uniprot.org/keywords/KW-0378) | Macromolecular complex, intracellular organelle, membrane-bounded organelle | Sustaining proliferative signaling; activating invasion and metastasis [35,36] |
| Q13177 | PAK2 | Metabolic process | [Kinase](http://www.uniprot.org/keywords/KW-0418), [serine/threonine-protein kinase](http://www.uniprot.org/keywords/KW-0723), [transferase](http://www.uniprot.org/keywords/KW-0808) | Organelle, intracellular | Sustaining proliferative signaling; resisting cell death [37] |
| Q14197 | ICT1 | Cellular process, organic substance metabolic process | Binding | [Mitochondrion](http://www.ebi.ac.uk/QuickGO/GTerm?id=GO:0005739) | Resisting cell death [38] |
| Q96SB4 | SRPK1 | [Chromosome partition](http://www.uniprot.org/keywords/KW-0159), [differentiation](http://www.uniprot.org/keywords/KW-0221), [host-virus interaction](http://www.uniprot.org/keywords/KW-0945), [mRNA processing](http://www.uniprot.org/keywords/KW-0507), [mRNA splicing](http://www.uniprot.org/keywords/KW-0508) | [Kinase](http://www.uniprot.org/keywords/KW-0418), [serine/threonine-protein kinase](http://www.uniprot.org/keywords/KW-0723), [transferase](http://www.uniprot.org/keywords/KW-0808) | [Cytoplasm](http://www.uniprot.org/keywords/KW-0963), [endoplasmic reticulum](http://www.uniprot.org/keywords/KW-0256), [microsome](http://www.uniprot.org/keywords/KW-0492), [nucleus](http://www.uniprot.org/keywords/KW-0539) | Sustaining proliferative signaling; activating invasion and metastasis [39] |
| Q9H0R8 | GABARAPL1 | Metabolic and cellular process, biological regulation | [GABA receptor binding](http://www.ebi.ac.uk/QuickGO/GTerm?id=GO:0050811),  [beta-tubulin binding](http://www.ebi.ac.uk/QuickGO/GTerm?id=GO:0048487),  [protein binding](http://www.ebi.ac.uk/QuickGO/GTerm?id=GO:0005515) | Intracellular | Sustaining proliferative signaling; activating invasion and metastasis; resisting cell death; deregulating cellular energetics [40] |
| Q9UQ80 | PA2G4 | [rRNA processing](http://www.uniprot.org/keywords/KW-0698), [transcription](http://www.uniprot.org/keywords/KW-0804), [transcription and translation regulation](http://www.uniprot.org/keywords/KW-0805) | [Repressor](http://www.uniprot.org/keywords/KW-0678), [ribonucleoprotein](http://www.uniprot.org/keywords/KW-0687) | [Cytoplasm](http://www.uniprot.org/keywords/KW-0963), [nucleus](http://www.uniprot.org/keywords/KW-0539) | Resisting cell death [41] |
