# Supplementary material for: A Computational Strategy to Select Optimized Protein Targets for Drug Development toward the Control of Cancer Diseases
Source: PLoS One. 2015 Jan 27;10(1):e0115054. doi: 10.1371/journal.pone.0115054 (PMC4308075; doi:10.1371/journal.pone.0115054)
Supplement: S4 Table — (DOC) [file pone.0115054.s004.doc]

**Table S4**. Top-5 down-regulated genes and GO classification

| **Uniprotkb** | **Gene Name** | **Biological Process** | **Molecular Function** | **Cellular Component** | **Hallmarks of Cancer** |
| --- | --- | --- | --- | --- | --- |
| O14503 | BHLHE40 | Cellular macromolecule metabolic process, nucleic acid metabolic process, transport, cell cycle | Protein binding, catalytic, hydrolase and pyrophosphatase activity | Nuclear lumen, intracellular non-membrane-bounded organelle, cytoskeleton, lytic vacuolo, microbody | Resisting cell death; sustaining proliferative signaling; EMT process [42] |
| O43889 | CREB3 | Organic substance metabolic process, locomotion, adhesion, protein complex biogenesis, macromolecular complex assembly, protein complex subunit organization | Nucleic acid binding, organic cyclic compound binding, heterocyclic compound binding | Intracellular membrane-bounded organelle, cytoplasmic vesicle, membrane-bounded vesicle | Activating invasion and metastasis [43] |
| O95166 | GABARAP | Anatomical structure morphogenesis, developmental, protein modification and metabolic process, intracellular protein transport | Protein binding | Cytoplasmic part, intracellular membrane-bounded organelle | Activating invasion and metastasis [44] |
| P04083 | ANXA1 | Metabolic process, cell communication, anatomical structure development, transport, protein metabolic process, establishment of localization in cell | Phospholipase A2 inhibitor | Nuclear lumen, nuclear part | Tumor-promoting inflammation, sustaining proliferative signaling, resisting cell death [45] |
| P04406 | GAPDH | Transport, death, nitrogen compound metabolic process, cellular metabolic process | Protein binding | Intracellular membrane-bounded organelle, organelle lumen | Deregulating cellular energetics [17] |
| P04637 | TP53 | Cellular component biogenesis, cellular component organization | Binding | Intracellular organelle, intracellular, organelle | Deregulating cellular energetics, resisting cell death; sustaining proliferative signaling [46] |
| P08670 | VIM | Cellular developmental and metabolic process, response to stimulus, cellular component organization | Protein binding, transferase activity, transferring phosphorus-containing groups | Intracellular membrane-bounded organelle | Activating invasion and metastasis, EMT process [22] |
| P11021 | GRP78 | Signaling, cell communication, cellular response to stimulus | ATP, calcium ion, chaperone, enzyme, misfolded protein, protein, ribosome, ubiquitin protein ligase and unfolded protein binding, ATPase activity | Cytoplasmic, endoplasmic reticulum | Resisting cell death; sustaining proliferative signaling, avoiding immune destruction, Activating invasion and metastasis, inducing angiogenesis [47] |
| P25963 | NFKBIA | Cellular component organization or biogenesis | NF-kappaB, enzyme, nuclear localization sequence, protein, transcription factor and ubiquitin protein ligase binding | Intracellular non-membrane-bounded organelle | Sustaining proliferative signaling [48] |
| P31946 | YWHAB | Single-organism process, cellular process | Binding | Cytoplasm | Sustaining proliferative signaling, resisting cell death, activating invasion and metastasis [25] |
| P49841 | GSK3B | Biological rhythms, carbohydrate and glycogen metabolism, differentiation, neurogenesis, Wnt signaling pathway | Developmental protein, serine/threonine-protein kinase, signal transduction inhibitor, transferase | Cytoplasm, organelle | Sustaining proliferative signaling, resisting cell death [49] |
| P60520 | GABARAPL2 | Autophagy, protein transport, transport | ATPase, GABA, SNARE, beta-tubulin, microtubule and protein binding | Cytoplasmic vesicle, golgi apparatus | Resisting cell death [50] |
| P60709 | ACTB | [de novo posttranslational protein folding, ATP-dependent chromatin remodeling, Fc-gamma receptor signaling pathway involved in phagocytosis, adherens junction organization, blood coagulation, chromatin organization, innate immune response, protein folding](http://www.ebi.ac.uk/QuickGO/GTerm?id=GO:0051084) | ATP binding, Tat protein binding, kinesin binding, nitric-oxide synthase binding, structural constituent of cytoskeleton | Cytoskeleton, exosome, chromatin, nucleoplasm, protein and ribonucleoprotein complex | Activating invasion and metastasis [51] |
| P60953 | CDC42 | Differentiation, neurogenesis | Cell membrane, cytoplasm, cytoskeleton, membrane | Golgi membrane, cytoplasm, exosome, filopodium, microtubule organizing center, midbody, mitotic spindle, neuronal cell body, plasma membrane, secretory granule | Activating invasion and metastasis [52] |
| P63261 | ACTG1 | Fc-gamma receptor signaling pathway involved in phagocytosis, adherens junction organization, axon guidance, cellular component movement, innate immune response, membrane organization, sarcomere organization | ATP and protein binding, structural constituent of cytoskeleton | Cytoplasm, cytoskeleton | Activating invasion and metastasis [53] |
| Q71U36 | TUBA1A | de novo posttranslational protein folding, G2/M transition of mitotic cell cycle, cellular protein metabolic process, cytoskeleton-dependent intracellular, transport, microtubule-based process, protein folding, protein polymerization | GTP and protein binding, structural constituent of cytoskeleton, GTPase and structural molecule activity | Cytoplasm, cytoskeleton | Activating invasion and metastasis [54] |
| Q99959 | PKP2 | Cell adhesion | Intermediate filament, ion channel, protein kinase C binding, protein complex scaffold, sodium channel regulator activity | Cell junction, nucleus | Activating invasion and metastasis [55] |
| Q9H492 | MAP1LC3A | Autophagy, Ubl conjugation pathway | Phosphatidylethanolamine, phospholipid and protein binding | Cytoplasm, cytoplasmic vesicle, cytoskeleton, membrane, microtubule | Resisting cell death [56] |
