# Supplementary material for: A Computational Strategy to Select Optimized Protein Targets for Drug Development toward the Control of Cancer Diseases
Source: PLoS One. 2015 Jan 27;10(1):e0115054. doi: 10.1371/journal.pone.0115054 (PMC4308075; doi:10.1371/journal.pone.0115054)
Supplement: S5 Table — (DOC) [file pone.0115054.s005.doc]

**Table S5**. Therapeutic and/or *in vitro* compounds that show significant specificity in treatment

| **Uniprotkb** | **Gene Name** | **Drugs Name** | **Diseases** |
| --- | --- | --- | --- |
| O15264 | Mitogen-activated protein kinase 13 | MAPK13 inhibitors (compounds 61 and 62) *** [62] | Inflammatory airway diseases, including asthma and chronic obstructive pulmonary disease and cystic fibrosis |
| P00533 | Epidermal growth factor receptor | Gefitinib, erlotinib, cetuximab, lapatinib, panitumumab, vandetanib, trastuzumab, pertuzumab, afatinib, neratinib, AZD9291, CLO-1686* [63] | [Non-small cell lung](http://www.drugs.com/health-guide/non-small-cell-lung-cancer.html), pancreatic, colon, rectum, head and neck, breast, metastatic colorectal and thyroid cancer |
| P01106 | Myc proto-oncogene protein | JQ1 (NCT01587703), TMPyP4 [26]**, MYCN PNA conjugation with a somatostatin analog***, AVI-4126, a PMO targeting c-Myc*, TFOs***, TFOs covalently linked to the DNA-intercalating agent daunomycin*** [64] | Burkitt’s lymphoma, acute myeloid leukemia, neuroblastoma, prostate and breast cancer |
| P04183 | Thymidine kinase, cytosolic | siRNA*** [65] | Squamous carcinoma |
| P04406 | Glyceraldehyde-3-phosphate dehydrogenase | CGP 3466B** [66] | Motor neuron disease |
| P04626 | Receptor tyrosine-protein kinase erbB-2 | Trastuzumab, [pertuzumab](http://en.wikipedia.org/wiki/Pertuzumab), NeuVax™ vaccine* [67] | Breast cancer |
| P06748 | Nucleophosmin | NSC348884*** [68] | Prostate and colon cancer |
| P08238 | Heat shock protein HSP90-beta | siRNA*** [69] | Leukaemia |
| P08670 | Vimentin | Withaferin-A*** [70] | Sarcoma |
| P21860 | Receptor tyrosine-protein kinase erbb-3 | MM-121* [71] | Pancreatic ductal adenocarcinoma and ovarian carcinoma |
| P26641 | Elongation factor 1-gamma | siRNA*** [72] | Kidney normal cells |
| P30101 | Protein disulfide-isomerase A3 | siRNA*** [73] | Lung and renal carcinoma cells |
| P31946 | 14-3-3 protein beta/alpha | Difopein** [74] , R18*** [75] | Glioma and leukemia |
| P33993 | DNA replication licensing factor MCM7 | siRNA*** [76] | Prostate cancer |
| P38919 | Eukaryotic initiation factor 4A-III | siRNA*** [77] | Ullrich disease ﬁbroblasts |
| P51858 | Hepatoma-derived growth fator | HDGF-H3** [78] | Lung cancer |
| P52292 | Importin subunit alpha-1 | siRNA*** [79] | Epithelial ovarian carcinoma |
| P61326 | Protein mago nashi homolog | siRNA*** [77] | Ullrich disease ﬁbroblasts |
| P62993 | Growth factor receptor-bound protein 2 | CGP78850*** [80], C90** [81] | Breast, prostate and squamous cancer, glioblastoma and melanoma |
| P67870 | Casein kinase II subunit beta | siRNA*** [82] | Listeria monocytogenes |
| Q12873 | Chromodomain-helicase-DNA-binding protein 3 | siRNA*** [36,83] | Cervix adenocarcinoma |
| Q13177 | Serine/threonine-protein kinase PAK 2 | Staurosporine*** [84] | Bovine pulmonary artery endothelial (BPAE) cells |
| Q14197 | Peptidyl-tRNA hydrolase ICT1, mitochondrial | siRNA*** [38] | Cervix adenocarcinoma |
| Q96SB4 | Srsf protein kinase 1 | SRPIN340** [85] | Primary cells (e.g. podocytes), colon, prostate cancer, and melanoma |
| Q9H0R8 | Gamma-aminobutyric acid receptor-associated protein-like 1 | shRNA*** [40] | Breast cancer |
| Q9UQ80 | Proliferation-associated protein 2G4 | shRNA*** [86] ([Zhang Y](http://www.ncbi.nlm.nih.gov/pubmed?term=Zhang Y%5BAuthor%5D&cauthor=true&cauthor_uid=18355957) et al., 2008) | Breast cancer |

* Clinical trials; ** Pre-clinical animal models; *** *In vitro* assays; PNA - peptide nucleic acids; PMO - phosphorodiamidate morpholino oligomers; TFOs - phosphothioate stabilized triple helix forming oligonucleotides.
